# Large-Scale Evaluation of Quality of Care in 6 Countries of Eastern Europe and Central Asia Using Clinical Performance and Value Vignettes

**DOI:** 10.9745/GHSP-D-17-00044

**Published:** 2017-09-27

**Authors:** John W Peabody, Lisa DeMaria, Owen Smith, Angela Hoth, Edmond Dragoti, Jeff Luck

**Affiliations:** a QURE Healthcare, San Francisco, CA, USA.; b Department of Epidemiology & Biostatistics, University of California, San Francisco, School of Medicine, San Francisco, CA, USA.; c Department of Health Policy and Management, University of California, Los Angeles, School of Public Health, Los Angeles, CA, USA.; d The World Bank Group, Washington, DC, USA.; e Institute of Public Opinion Studies, Tirana, Albania.; f Faculty of Social Sciences, Tirana University, Tirana, Albania.; g College of Public Health and Human Sciences, Oregon State University, Corvallis, OR, USA.

## Abstract

**Background:**

A significant determinant of population health outcomes is the quality of care provided for noncommunicable diseases, obstetric, and pediatric care. We present results on clinical practice quality in these areas as measured among nearly 4,000 providers working at more than 1,000 facilities in 6 Eastern European and Central Asian countries.

**Methods:**

This study was conducted between March 2011 and April 2013 in Albania, Armenia, Georgia, Kazakhstan, Kirov Province in Russia, and Tajikistan. Using a probability proportional-to-size sampling technique, based on number of hospital beds, we randomly selected within each country 42 hospitals and their associated primary health care clinics. Physicians and midwives within each clinical area of interest were randomly selected from each hospital and clinic and asked how they would care for simulated patients using Clinical Performance and Value (CPV) vignettes. Facility administrators were also asked to complete a facility survey to collect structural measures of quality. CPV vignettes were scored on a scale of 0% to 100% for each provider. We used descriptive statistics and *t* tests to identify significant differences in CPV scores between hospitals and clinics and rural vs. urban facilities, and ANOVA to identify significant differences in CPV scores across countries.

**Results:**

We found that quality of care, as concurrently measured by performance on CPV vignettes, was generally poor and widely variable within and between countries. Providers in Kirov Province, Russia, had the highest overall performance, with an average score of 70.8%, while providers in Albania and Tajikistan had the lowest average score, each at 50.8%. The CPV vignettes with the lowest scores were for multiple noncommunicable disease risk factors and birth asphyxia. A considerable proportion (11%) of providers performed well on the CPV vignettes, regardless of country, facility, or structural resources available to them.

**Conclusions:**

Countries of Eastern Europe and Central Asia are challenged by poor performance as measured by clinical care vignettes, but there is potential for provision of high-quality care by a sizable proportion of providers. Large-scale assessments of quality of care have been hampered by the lack of effective measurement tools that provide generalizable and reliable results across diverse economic, cultural, and social settings. The feasibility of quality measurement using CPV vignettes in these 6 countries and the ability to combine results with individual feedback could significantly enhance strategies to improve quality of care, and ultimately population health.

## Abstract (precis)

When providers in 6 different countries were asked how they would care for the same patient, there was wide variation within and between countries. Nevertheless, 11% of the physicians scored over 80%, suggesting good quality of care is possible even with resource constraints. Use of validated clinical vignettes, which can be applied affordably at scale, could help improve quality of services in low- and middle-income countries.

## INTRODUCTION

Eastern Europe and Central Asia (ECA), which encompasses 30 countries, is diverse both culturally and linguistically, with little but geography tying them together.^1^ Following the breakup of the Soviet Union, ECA health systems faced severe challenges. Scarce resources, weak governance structures, and a lack of accountability inherited from the past have plagued efforts to improve access, quality, and efficiency. Social and economic upheavals associated with transition have compounded the challenges.^2^ Since 2000, substantial investments to improve health care access in the region have focused on rebuilding and restoring health facilities.^1^^,^^3^^,^^4^ Despite investments, poor quality of care has been entrenched, frustrating obvious opportunity to rapidly improve the health status of the ECA population.^2^^,^^4^

A significant determinant of population health outcomes is the quality of care provided specifically for noncommunicable, obstetric, and pediatric services.^5^^–^^8^ Access to and use of high-quality primary care prevents and reduces development of noncommunicable diseases and associated complications following an acute illness.^9^ Similarly, high-quality intrapartum and perinatal care decreases incidence of postpartum bleeding and puerperal and neonatal sepsis.^10^^–^^12^ Improving quality—and, in turn, health outcomes—yields at least 2 important economic benefits. First, better health among the working population improves productivity and reduces dependency burden on families.^13^ Second, reduced government and private spending for avoidable acute care, as well as for disability arising from avoidable disease, frees resources that can be allocated for education and other productive investment.^14^^,^^15^

Available evidence indicates that worldwide quality of clinical care services—what providers do when they see a patient—is often poor as measured against evidence-based standards and varies widely between and within countries, as well as between and within clinics and hospitals.^13^^,^^16^^,^^17^ Adequately trained and motivated clinical providers, even if they have only basic equipment and supplies, can offer high-quality care for a wide range of acute and chronic diseases,^18^ but to be of use, the delivery of quality care must be *measured.* Care quality, moreover, can be improved in developing and emerging countries in a short period of time, and by the existing workforce, without massive investments in new facilities or human capital.^19^ Nevertheless, a dearth of data exists on clinical practice quality in many parts of the world, especially in the ECA region.^5^^,^^20^

In 2011, the World Bank undertook an ambitious cross-national and intra-country analysis of the quality of care in 6 ECA countries: Albania, Armenia, Georgia, Kazakhstan, Kirov Province in Russia, and Tajikistan. Similar to other projects of this scale,^21^^–^^24^ the purpose of this study was to produce national and cross-national comparative quality of care data. This was accomplished using Clinical Performance and Value (CPV) vignettes, which are simulations of clinical scenarios, to measure the quality of clinical services—sometimes referred to as care processes—among 3,584 doctors and 384 midwives in 1,039 facilities from 391 hospitals and 648 associated primary health care (PHC) clinics. The findings presented in this article show a comprehensive concurrent evaluation of quality provided in the ECA region and provide policy makers with insights into where quality improvement is needed. The results also identify to hospitals and facilities who, in the aggregate, is providing the best (or worst) care and can give individual doctors and midwives an opportunity to evaluate and improve their own practice through individualized feedback.

In 2011, the World Bank undertook a cross-national analysis of quality of care in 6 Eastern European and Central Asian countries.

CPV vignettes are simulations that have been uniquely validated against standardized patients. The vignettes can be administered on paper or electronically. Each provider is presented with the same case, or vignette, and asked to take the (simulated) patient's history, do an examination, order the necessary tests, make a diagnosis, and specify a treatment plant—thereby simulating a patient visit and providing an opportunity to evaluate the physician's knowledge and care processes. Other, more limited types of vignettes have not always corresponded well to actual clinical practice,^25^^,^^26^ prompting us to develop CPV vignettes. The CPV vignette, compared with other vignettes, uses open-ended questions and flexible multistage evaluations to simulate actual practice and actual patient visits to validate their accuracy.^27^^,^^28^

As a tool, CPV vignettes have been shown to outperform both chart abstraction (medical record review), a common measurement method in some settings,^27^^,^^29^^,^^30^ and direct observation, which is often used in developing countries.^25^^,^^31^ Chart abstraction can be more costly than CPVs because it requires records to be found and secured, and abstractors to review and record details from each clinical visit.^28^ Clinical charting is also highly variable in different countries, obviating the possibility of using abstraction in cross-national studies.^32^ By contrast, CPVs with explicit criteria can be scored very rapidly, and no adjustment for case-mix variation is needed. Direct observation, unlike CPVs, is influenced by the Hawthorne effect, wherein a provider's actions change due to the observation. Interestingly, recent research suggests that this observational bias may disappear over time.^33^ CPVs have been shown to correlate well with actual physician practice and have been deployed at an affordable cost in a variety of clinical practice settings around the world.^6^^,^^8^^,^^30^ In our other work, we have shown program costs for administering CPVs at US$2.25 per program beneficiary.^34^ Even when abstraction, direct observation, or standardized patients can be implemented, the data need to be case-mix adjusted. CPV vignettes thus allow for direct comparison of provider performance within and between countries, both individually and in the aggregate, and were therefore used to measure clinical practice in this study.

Clinical Performance and Value (CPV) vignettes, which are simulations of client scenarios, have been shown to outperform both chart abstraction and direct observation as a tool to measure quality of care.

## METHODS

### Setting

Between March 2011 and April 2013, the World Bank conducted a large, comprehensive cross-national and intra-country analysis of the quality of care among 6 ECA countries where it was working on quality of care projects (Albania, Armenia, Georgia, Kazakhstan, Kirov Province in Russia, and Tajikistan).^1^ While these 6 countries are diverse, they may not capture the full diversity of the region. Notwithstanding, the health care systems of these countries have a wide variety of organizational structures, financial and human resources, and health priorities and outcomes.^35^ Population and health characteristics of the 6 countries are listed in Table 1.

**TABLE 1.** Population and Health Characteristics of the ECA Countries Included in the Quality of Care Study, 2013^a^

	Albania	Armenia	Georgia	Kazakhstan	Kirov Province, Russia^b^	Tajikistan
Population	3,173,000	2,977,000	4,341,000	16,441,000	1,315,003	8,208,000
World Bank income group	Upper middle	Lower middle	Lower middle	Upper middle	High (Russia)	Low
% of population living in urban areas	53%	63%	53%	53%	74% (Russia)	27%
Life expectancy at birth	73 male 76 female	67 male 75 female	71 male 78 female	63 male 73 female	64 male 76 female	68 male 70 female
Total expenditure on health as % of GDP (2012)	6.0%	4.5%	9.2%	4.2%	6.3% (Russia)	5.8%
No. of physicians per 1,000 population	1.2	3.8	4.8	4.1	4.7	2.1
Neonatal mortality (deaths per 1,000 live births)	7	8	8	8	5	21
Maternal mortality (deaths per 100,000 live births) (2013)	21	29	41	26	24 (Russia)	44
Adult risk factors						
Tobacco smoking (2011)	26%	22%	27%	24%	40% (Russia)	N/A
High blood pressure (2008)	37%	42%	43%	35%	38% (Russia)	31%
Obesity (2008)	21%	24%	22%	24%	27% (Russia)	9%
% of total deaths due to NCDs, all ages, both sexes (2013)	89%	92%	93%	84%	86% (Russia)	62%
% of total deaths due to cardiovascular disease, all ages, both sexes (2013)	59%	54%	69%	54%	60% (Russia)	38%

Abbreviations: ECA, Eastern Europe and Central Asia; GDP, Gross Domestic Product; NCDs, noncommunicable diseases; UNICEF, United Nations Children's Fund; WHO, World Health Organization.

Sources: Albania, WHO (http://www.who.int/countries/alb/en/); Armenia, WHO (http://www.who.int/countries/arm/en/); Georgia, WHO (http://www.who.int/countries/geo/en/); Kazakhstan, WHO (http://www.who.int/countries/kaz/en/); Russia, Knoema World Data Atlas (http://knoema.com/atlas/Russian-Federation/Kirov-Region/Population); Tajikistan, WHO (http://www.who.int/countries/tjk/en/); and neonatal mortality for all countries, UNICEF (http://data.unicef.org).

^a^ 2013 data unless otherwise specified.

^b^ Data for Kirov Province only, unless otherwise specified.

### Field Operations

For this study, we assembled a cross-national research team, consisting of academicians, country programmatic experts, survey firms, and a private firm with expertise in the measurement of quality using CPV vignettes (QURE). The World Bank facilitated country-level buy-in and support for the study via local representatives and the Ministry of Health (MOH). Local MOH personnel constructed the sample frame rosters of hospitals, clinics, and providers. We secured signed letters of support from the MOH to facilitate access to the study sites and review of the data collection instruments. Local firms in each of the countries carried out the fieldwork and primary data collection.

Two regional training sessions for the data collection teams were conducted. In November 2011, a 3-day training session was held in Tbilisi, Georgia, with data collection firms from Armenia, Georgia, and Tajikistan. Trainees were schooled in standardizing data collection and were given an orientation to the CPV methodology and procedures. In addition, all the instruments were piloted at a local hospital with the trainers present. Fieldwork for these countries took place from January to March of 2012.

The second training session was held in Tirana, Albania, in May 2012, before the launch of the study in Albania, Kazakhstan, and Russia. Data collection for Albania and Russia was carried out from June to September 2012, and for Kazakhstan between January and April 2013.

All field teams consisted of 1 supervisor and 2 or 3 enumerators. With the exception of Kazakhstan, which had real-time data entry through laptops, data collection was conducted on paper instruments and completed by the enumerators that were then sent to the central office of each country for data entry. Providers' responses to the CPV vignettes were translated from the local language to English. Electronic files with each of the completed CPV responses were sent to QURE for scoring.

Supervision during the entire data collection process was carried out by QURE and supported by the World Bank through weekly teleconference meetings to monitor progress, address issues that arose in the field, and review data quality.

### Sampling Methods

*Facilities*. PHC centers and secondary referral hospitals provide the majority of NCD, neonatal, and obstetric care—the focus of our study—in each country. Local survey firms compiled rosters of every hospital in each country, including number of beds, rural/urban designation, whether the hospital attended births, and whether it was a single-specialty hospital. Hospitals with fewer than 10 beds or that did not provide internal medicine care were excluded.

We used a probability proportional-to-size sampling technique, based on number of hospital beds, and randomly selected 42 hospitals per country. A census sample was conducted in Albania and Kirov Province, Russia, because they had fewer than 42 hospitals overall. Eight selected hospitals in Armenia and 7 in Tajikistan did not have maternity services, so the geographically closest maternity hospital was also included in the study. Surveyors visited every randomly selected hospital to confirm the inclusion criteria and willingness to participate. In Armenia, 2 private hospitals refused to participate, but no other hospital refusals occurred.

From the study hospitals, a comprehensive list of associated PHC clinics was generated. PHC clinics included polyclinics, general medicine clinics, and health care outposts. For each hospital, we randomly selected 3 associated PHC clinics for participation.

We randomly selected hospitals and primary health care clinics for inclusion in the study.

*Providers*. Physician and midwife providers were selected from the final hospital and PHC rosters for each country. Physicians were classified by service line—internal medicine/general practice, pediatrics, obstetrics, or specialty care. We know that the minimum clinically meaningful difference is 3% to 4% in CPV scores.^8^^,^^36^ At this effect size, a total sample of 3,830 observations was required to distinguish differences between countries, and within each country by rural vs. urban setting, provider specialties, and among facility types for each of the three disease areas. We, therefore, randomly selected 4 physicians at the service line level in each hospital, along with 3 midwives at the hospital level and 3 primary care providers in the clinics, to generate a representative sample for each provider group.

### Epidemiology and Disease Selection

The study focused on the quality of care in 3 clinical areas: care of the noncommunicable (chronic) disease (NCD) patient, care of the newborn, and care of the mother. These clinical areas encompass both ambulatory and nonambulatory settings.

The study focused on quality of care in 3 clinical areas: noncommunicable diseases, neonatal care, and obstetric care.

Chronic disease is a well-documented contributor to the ECA region's disease burden.^37^^,^^38^ Mortality from major chronic NCDs in Central and Eastern Europe is almost twice that of European Union countries and afflicts a younger age group.^39^ High rates of chronic disease also have an increasingly negative impact on the labor supply, including workforce participation, hours worked, wages, and earnings.^40^

Neonatal mortality comprises 38% of all under-5 mortality worldwide,^41^ and is high in the ECA region, particularly in Central Asia where perinatal and neonatal mortality rates are 5 times higher than in Western Europe.^42^ An estimated 90% of neonatal deaths worldwide are caused by birth asphyxia, infections, or complications of prematurity.^43^ Although it is widely believed improving neonatal health requires access to sophisticated technology, the majority of neonatal deaths can be avoided with low-cost, evidence-based care.^41^ As a result, very basic quality interventions have a major impact on neonatal outcomes.^42^

Maternal health remains at the forefront of global health efforts, and postpartum hemorrhage is a leading cause of maternal mortality during childbirth. Maternal mortality is particularly high in Tajikistan and Georgia (Table 1), with little prior research focused on provider quality of maternal health care in the ECA region. Most studies found in this area have focused more on systemic quality of care rather than provider quality of care. This emphasis on systemic care is underscored by a recent systematic review on the barriers to accessing adequate maternal care in Central and Eastern Europe, which found a total of 21 articles that looked at improvements in maternal care.^44^ Of these 21 articles, only 7 examined the appropriateness of maternal care, all of which agree that there was a lack of needed skills in delivering care.

### Quality Measurement

#### Framework

This study used the structure-process-outcome framework to measure quality.^45^ Health *outcomes*, such as disability or mortality, are the ultimate impact of health policy but some of these outcomes are challenging to measure accurately and hard to causally distinguish between disease severity and the quality of the health care services.^46^ Care *processes*, or care services that patients receive from health care providers, are proximate to outcomes, occur *every* time there is a patient visit, and thus are potentially an ideal measure of quality.^41^ *Structural* factors, such as provider training and facility characteristics, are perhaps the most readily measured but have much less direct impact on health outcomes, being mediated by the process of care delivered by the provider.

This study collected data on the quality of care using CPV vignettes to measure clinical practice, or care processes, for the 3 disease areas of interest. Additionally, data were collected in each country on the structural measures of quality in health care facilities where the providers practiced.

#### Measurement of Care Processes Through CPV Vignettes

A CPV vignette is a proprietary quality measurement tool designed to test a provider's ability to provide the proper care and treatment of simulated patients. In this study, each CPV vignette is a paper-based simulated case that starts with a typical patient presenting with symptoms and signs of an undisclosed clinical condition. By the nature of the case simulation, variation introduced by case-mix is removed, thereby allowing for direct comparison of provider performance within and between countries, both individually and in the aggregate. In addition, CPVs are designed to simulate a complete clinical encounter, making it possible to assess a provider's clinical decisions from when a patient enters to when a patient leaves.

For this study, physicians and midwives were required to respond in writing to open-ended questions indicating how they would normally gather information in their own settings to solve the case, replicating what they would do in a real-life scenario. The respondents had to answer questions on 5 aspects of the care process:

1. Taking the patient's history
2. Doing the physical examination
3. Requesting (and receiving) radiological or laboratory tests
4. Making a diagnosis
5. Prescribing disease-specific treatment

The CPV vignettes took into account particular capacity limitations in each country. For example, availability of chest CTs is limited throughout Kazakhstan, so this test is not an option in the CPV vignettes for these providers.

Using the CPV vignettes, providers indicated how they would normally gather information in their own settings to solve the case, from taking the patient's history to prescribing treatment.

The study investigators created 5 CPV vignettes, initially in English, for the 3 disease areas of interest: NCD (including a multiple NCD risk factor case and an acute myocardial infarction [AMI] case), neonatal care (pneumonia and birth asphyxia cases), and obstetrics (postpartum hemorrhage case). The midwives were assessed only for the postpartum hemorrhage case. In developing these cases, WHO guidelines were used as the criteria for measuring quality of care for all cases within each health system.^47^^,^^48^ Guidelines from relevant European medical societies were also added to increase local relevancy and buy-in from the MOHs.^49^

Every case required the clinician to perform a thorough history and physical examination of the patient. The information gleaned from these 2 domains then informed the next steps (ordering laboratory tests and images, or doing a procedure) that the clinicians felt they needed to take to reach the correct diagnosis. Once a diagnosis (correct or incorrect) was reached, the clinician then formulated a treatment plan (e.g., counseling, medications, a procedure) as well as the follow-up for the patient (Box).

BOX **Sample Clinical Performance and Value Vignette**

In a typical Clinical Performance and Value (CPV) vignette, providers are given a presenting problem for a simulated patient. For example:

*Presentation*: *Selim, a 12-day-old newborn boy, is brought to your clinic by his mother because he is feeding poorly, won't sleep, and is irritable.*

The providers are then asked what questions they would ask the patient (or in this case, his mother) about his history. The following is Selim's history, given to the providers once they have asked all their questions:

Full History: According to Selim's mother, he had fair suck, good cry, and good activity when he was brought home from the hospital 9 days ago. Yesterday, he became irritable, started crying all the time, and began coughing and refusing his feedings. This morning, he had a moderate- to high-grade undocumented fever. The mother reports Selim has not had any seizures, a rash, or excess sleepiness. His stools were of normal consistency although the frequency has decreased from 2 to 3 times each day to once daily. He had 5 diaper changes yesterday, all of which were fully soaked, according to the mother. There were no episodes of vomiting, seizures, jaundice, lethargy, or increased sleeping time. He is exclusively breastfed.

Selim was born via primary low-segment cesarean delivery to a 22-year-old primigravida. The length of gestation was 36 weeks by dates, confirmed with an earlier ultrasound. The obstetrician ruptured the bag of water artificially at the 5th hour of labor and the meconium was clear. The decision to perform a cesarean delivery was made after 7 hours of labor due to some decelerations noted on intrapartum fetal monitoring. At birth, his Apgar score was 7, improving to 9, and he was a term baby by pediatric aging (37 weeks). He weighed 3.2 kg at birth.

His mother had 3 prenatal consults obtained in the polyclinic in her oblast [administrative division]. There were no blood pressure elevations during the pregnancy, except during labor. She did not undergo testing for gestational diabetes. She did not report any recent cough/colds, fever, dysuria, vaginal discharge, or vaginal bleeding. The mother admits she had smoked cigarettes intermittently during the pregnancy, but there was no alcohol or illicit drug use.

She denies any history of sexually transmitted infections. She is up-to-date with her tetanus shot but received only 1 dose of the hepatitis B vaccine. She has never been hospitalized and is on no medications. She has no known drug allergies.

Next, the providers are asked which physical examinations they would perform. In Selim's case, the following information would be given to the providers after their response.

Physical Examination: On examination of the infant, he appears acutely ill and obviously irritable. There are no rigors or tremors noted. The respiratory rate is 90/min and the heart rate is 160/min. The rectal temperature is 38.8° C. Weight is 3 kg (down from 3.2 kg at birth). Breathing is shallow. There are no retractions, grunting, or cyanosis. On auscultation, the breath sounds were vesicular with no rales, wheezing, or crackles. There were no cardiac murmurs heard. The abdomen was not distended, and bowel sounds were present. The anterior fontanel was intact and slightly bulging, particularly when the infant cries. Capillary refill time was 2 seconds. Oxygen saturation was 87% on room air.

Then, the providers are asked which imaging or laboratory tests they would order to aid their diagnosis. Depending on what they ordered, they would get the following test results for Selim:

- Complete blood count: normal hemoglobin of 12.8 g/dL and hematocrit (0.42); white blood cell (WBC) count is 13.8 x 10^9^/L with a predominance of segmented PMNs (polymorphonuclear leukocytes) (84%) and 12% bands; platelets are 107.
- Glucose: 4.1 mmol/L.
- Blood culture: drawn and are pending.
- Chest x-ray: showed patchy infiltrates over both lung fields.
- Lumbar tap: yielded a turbid cerebrospinal fluid with a:
  - Low glucose level (1.9 mmol/L)
  - Elevated protein content (160 mg/dL)
  - Pleiocytosis (WBC count 265 cells/mm^3^ with predominance of polymorphonuclear cells).
  - Gram stain of the cerebrospinal fluid (CSF) revealed the presence of gram-negative coccobacilli.
  - CSF culture: result pending
- Serum electrolytes and creatinine: normal.

At this stage, the providers are asked what their diagnosis is.

Selim's diagnosis: Neonatal sepsis with pneumonia and meningitis, moderate to severe.

Finally, providers are asked to delineate the next steps in the patient's treatment plan.

*Selim's treatment plan:*

- Admit to hospital.
- Supplemental oxygen by face mask, monitor oxygen saturation.
- Intravenous glucose 10% in 0.18 normal saline.
- Intravenous antibiotics: ampicillin plus aminoglycoside OR intravenous aminoglycoside plus expanded spectrum penicillin antibiotic (or equivalent).
- Monitor vital signs including oxygenation.
- Monitor occipitofrontal circumference (to detect hydrocephalus).
- Repeat blood cultures after 24–48 hours.
- Repeat lumbar puncture after 24–48 hours of initiating antibiotics to document sterilization of CSF.

Through the CPV vignette, we can assess the process of care practitioners would provide and how that process might lead to different outcomes for the simulated patient. For example, if Selim would have seen Physician A from our study, he would have seen a doctor who thoroughly explored his history, asking not only about the current episode prompting his mother to bring him to the clinic but also about his birth history details, and taken a detailed physical examination. These actions led Physician A to order a CBC, blood culture, glucose, and spinal tap; the spinal tap led to the discovery of gram-negative coccobacilli and Physician A's diagnosis of neonatal sepsis with pneumonia and meningitis. With this diagnosis, Physician A indicated she would immediately admit Selim to the hospital and recommended a full treatment plan.

On the other hand, if Selim would have seen Physician B from our study, he would have encountered a doctor who explored only the current presentation of the newborn without asking about any prior history. Physician B ordered only a CBC, blood culture, and chest x-ray; the x-ray showed patchy infiltrates in both lungs, leading the physician to diagnose Selim with community-acquired pneumonia. Fortunately, Physician B felt the pneumonia was serious enough to require immediate IV antibiotics and hospitalization, but without the proper workup to make the right diagnosis, Selim would not have received the follow-up blood culture, lumbar puncture, and adequate monitoring warranted in a newborn this sick.

For details on how this sample CPV vignette was scored for Physician A and Physician B, see the Supplement.

Each of the developed cases in this study had at least 1 essential element for the clinician to identify and treat. The birth asphyxia cases required the provider to anticipate and provide the necessary care for these newborn patients (e.g., gasping respirations requiring bag-and-mask ventilation). The neonatal pneumonia cases required proper workup (recognition of tachypnea and decreased alertness that should lead to blood cultures, chest x-ray, and lumbar puncture) to not only identify the pneumonia but also exclude the possibility of sepsis and meningitis. The maternal postpartum hemorrhage cases looked at whether the clinicians recognized tachycardia and hypotension in a multigravida patient, requiring an evaluation of uterine blood loss and surgical curettage. The AMI cases required recognition of the acuity, confirmation of this with either troponin or creatine kinase (CK)-MB levels, and provision of comprehensive pharmacologic ischemic interventions.

To ensure that the CPV vignettes were appropriate for each local setting while retaining comparability across countries, the patient narrative was adjusted to the specific country or region, for example, by changing pseudonyms for patients and adapting social characteristics for each country. All study instruments, including the CPVs, were translated into the local language as well as Russian and then piloted to ensure clarity. They were then back-translated into English to check for fidelity with the original instruments. All study instruments were also reviewed in detail by MOH representatives to ensure that the questions and the CPV cases presented were relevant to their particular setting.

The data collection in Albania and Russia was conducted in the main language of the country (Albanian and Russian, respectively). For the remaining countries, providers had the option to complete the questionnaire and CPV either in the national language (Georgian, Tajik, Armenian, or Kazak) or in Russian.

#### Structural Measures of Quality

A facility survey was completed by an administrator at each hospital and primary care site. The facility survey collected structural quality data about personnel, material and financial resources, and clinical services provided. Other collected structural measures included management approaches, available equipment (54 items), laboratory tests (35 items), and pharmacy (43 medications).

### Data Analysis

The CPV vignettes for each provider were scored on a scale of 0% to 100%, where 100% indicated perfect conformity to the recommended clinical practice. To ensure that items of lesser importance were not equally weighted with items of greater importance, lesser items were grouped together into a single item. Providers receiving a “standard practice” rating were those who scored within 1 standard deviation of the mean of all providers, while above average was anything above the mean and substandard was anything below the mean. Previous studies have shown that a 3% increase or difference in absolute scores is clinically meaningful, and any score above 80% indicates delivery of high-quality care for the specific clinical scenarios tested.^6^

We used descriptive statistics and *t* tests to identify significant differences in CPV scores between hospitals and polyclinics and rural vs. urban facilities, and ANOVA to identify significant differences in scores across countries. For structural measures of quality, we performed descriptive statistics. All statistical analyses used STATA version 12.1 (StataCorp, College Station, Texas, USA).

### Ethical Review

Ethical approval for this study was acquired in accordance with each participating country's MOH, who determined that since this was a survey on the quality of clinical care provided, an Institutional Review Board (IRB) review within each country was unnecessary. Informed consent was obtained in writing from all physician and midwife participants; there were no patient-level data involved; and the analysis was done anonymously. The names of the providers and hospitals were changed to numerical identifiers on the completed vignettes before they were scored. The study protocol was formally reviewed by the Chesapeake IRB, which determined that the protocol was exempt from review under the United States Code of Federal Regulation, 45 CFR 46.

## RESULTS

### Background Characteristics

In total, 3,584 randomly sampled physicians and 384 randomly sampled midwives completed the surveys and CPVs across the 6 countries. The average age of the physicians was 46.2 years, with country averages ranging from 41.8 years in Kazakhstan to 50.1 years in Georgia. Midwives were, on average, 43.9 years of age, with a high of 48.2 years in Georgia and a low of 39.2 years in Kazakhstan. Over two-thirds (71.2%) of the physicians and nearly all (98%) of the midwives were women.

### CPV-Measured Quality: Cross-Country Comparisons

The average CPV vignette score varied between the highest-performing and lowest-performing country by 20 percentage points. Providers in Kirov Province, Russia, had the highest overall performance with an average vignette score of 70.8%, followed by providers in Kazakhstan, Georgia, and Armenia, with country-level scores of 64.1%, 63.2%, and 61.0%, respectively. At the lowest end, providers in Albania and Tajikistan each had an average score of 50.8%. This country-level variation persisted across clinical areas (Figure).

**FIGURE:**
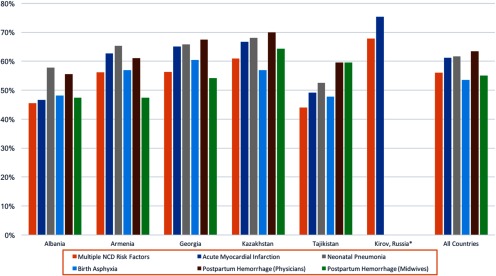
Mean CPV Vignette Scores by Country and Condition Abbreviations: CPV, Clinical Performance and Value; NCD, noncommunicable disease. *Russia measured quality of care only for NCDs.

Providers in Kirov Province, Russia, had the highest overall performance on the CPV vignettes, while those in Albania and Tajikistan had the lowest.

Physicians in Russia (Kirov Province) and Kazakhstan typically provided the highest quality of care overall as measured by the CPV vignettes. Tajikistan performed lower on the CPV vignettes than any of the other countries (*P*<.01).

For neonatal care, with scores ranging between 60% and 68%, providers in Kazakhstan, Georgia, and Armenia performed significantly better than those in Tajikistan (*P*<.05). (Russia measured quality only for NCDs.) Providers overall struggled more with the birth asphyxia case (53.6% average score across all countries) than the neonatal pneumonia case (61.7% average score) (Table 2) (*P*<.01).

**TABLE 2.** Variation of Mean CPV Vignette Scores (%) for Neonatal Care Conditions, By Country

	Neonatal Pneumonia				Birth Asphyxia			
	Mean	25th Percentile	75th Percentile	Difference Between 25th and 75th Percentiles	Mean	25th Percentile	75th Percentile	Difference Between 25th and 75th Percentiles
**All Countries**	**61.7**	**51.7**	**75.0**	**23.3**	**53.6**	**42.3**	**65.1**	**22.8**
Albania	57.8	47.5	72.5	25.0	48.1	38.0	57.5	19.5
Armenia	65.3	55.8	77.5	21.7	57.0	47.5	70.0	22.5
Georgia	65.9	57.5	77.5	20.0	60.4	47.5	72.5	25.0
Kazakhstan	68.0	60.0	77.5	17.5	56.9	44.6	70.5	25.9
Kirov, Russia	N/A	N/A	N/A	N/A	N/A	N/A	N/A	N/A
Tajikistan	52.6	42.5	65.0	22.5	47.7	37.5	59.9	22.4

Abbreviation: CPV, Clinical Performance and Value.

Among physicians, the best performers with the postpartum hemorrhage CPV vignette were from Kazakhstan (69.9% average score) and Georgia (67.4%), while for midwives, those from Kazakhstan (64.3%) and Tajikistan (59.6%) provided the highest quality of care as measured by the CPV vignettes (Table 3). In general, obstetrician-gynecologists scored higher than general practice physicians (about 64% vs. 58%, respectively) on postpartum hemorrhage. In Tajikistan, midwives performed similarly to physicians (about 60% average score), in contrast to the other countries where the physicians provided higher CPV-measured quality of care.

**TABLE 3:** Variation of Mean CPV Vignette Scores (%) for Postpartum Hemorrhage, By Country and Type of Clinician

	Physicians				Midwives			
	Mean	25th Percentile	75th Percentile	Difference Between 25th and 75th Percentiles	Mean	25th Percentile	75th Percentile	Difference Between 25th and 75th Percentiles
**All Countries**	**63.5**	**52.9**	**75.2**	**22.3**	**55.1**	**43.6**	**67.1**	**23.5**
Albania	55.6	47.5	63.1	15.6	47.4	35.0	59.4	24.4
Armenia	61.1	52.3	70.6	18.3	47.4	34.9	59.5	24.6
Georgia	67.4	56.3	77.5	21.2	54.2	44.5	63.8	19.3
Kazakhstan	69.9	61.5	79.4	17.9	64.3	56.1	74.8	18.7
Kirov, Russia	N/A	N/A	N/A	N/A	N/A	N/A	N/A	N/A
Tajikistan	59.5	48.7	72.6	23.9	59.6	48.9	71.4	22.5

Abbreviation: CPV, Clinical Performance and Value.

Across all regions in this study, NCD and neonatal care performance was higher at hospitals than at primary care facilities, although this difference was not significant (*P*>.05).

### CPV-Measured Quality: Within-Country Comparisons

While between-country CPV score averages varied by 20 percentage points, variation in quality of care *within* countries, as measured by the CPV vignettes, was much greater. But in each country—regardless of the clinical setting studied, local resource constraints, and other challenges facing providers—many individual practitioners performed well. Using a threshold CPV score of 80%, 11% of the providers demonstrated this high level of care or higher. Among these high performers, 87% were specialists and 13% were general practice or internal medicine physicians. In Table 4, the 25th percentile of performance for treatment of an AMI episode overall was only 48.8%, meaning 1 in 4 physicians performed at or below this level. Kirov, Russia, had the least variability of care on both the AMI and multiple NCD risk factor vignettes, with one-half of its physicians performing between 65% and 85% on the AMI vignette and between 62% and 73% on the multiple NCD risk factor vignette. Similarly alarming findings can be found in Table 2 and Table 3, where looking at poor performance, we found one-quarter of all providers scored below 50%.

**TABLE 4.** Variation of Mean CPV Vignette Scores (%) for Noncommunicable Disease Conditions, By Country

	CPV Vignette Condition							
	Acute Myocardial Infarction				Multiple NCD Risk Factors			
	Mean	25th Percentile	75th Percentile	Difference Between 25th and 75th Percentiles	Mean	25th Percentile	75th Percentile	Difference Between 25th and 75th Percentiles
**All Countries**	**61.1**	**48.8**	**74.7**	**25.9**	**56.0**	**44.9**	**67.1**	**22.2**
Albania	46.7	36.2	57.3	21.1	45.5	37.0	53.1	16.1
Armenia	62.7	53.3	73.9	20.6	56.1	44.3	67.1	22.8
Georgia	65.0	54.0	76.6	22.6	56.3	47.1	67.0	19.9
Kazakhstan	66.7	55.8	78.7	22.9	61.0	53.0	69.6	16.6
Kirov, Russia	75.3	65.1	84.5	19.4	67.8	62.3	72.5	10.2
Tajikistan	49.2	38.3	58.1	19.8	44.0	35.6	54.3	18.7

Abbreviations: CPV, Clinical Performance and Value; NCD, noncommunicable disease.

11% of providers across all 6 ECA countries performed at a high level on the CPV vignettes.

### CPV-Measured Quality by Provider Characteristics

Bivariate analysis showed female clinicians had significantly higher CPV scores than male clinicians (60.9% vs. 52.9%, respectively; *P*<.01). At the physician level, specialists (62.5%) performed significantly better than general practice physicians (62.5% vs. 55.3%, respectively; *P*<.01). This finding held within individual CPV case types (Table 5). For example, general practitioners scored 12.1 percentage points lower than cardiologists for AMI cases. In general, within a specific disease area, those with training in the specialty of the case scored higher (with scores by case type ranging from 56.2% to 69.2%) than their general practice colleagues (48.5% to 59.0%) (*P*<.01). One exception was the multiple NCD risk factor case where internal medicine physicians scored slightly ahead of cardiologists (58.7% vs. 55.9%, respectively).

**TABLE 5.** Average CPV Vignette Scores (%), by Clinician Specialty and CPV Vignette Condition

	CPV Vignette Condition				
	Multiple NCD Risk Factors (n=1,034)	Acute Myocardial Infarction (n=1,027)	Neonatal Pneumonia (n=733)	Birth Asphyxia (n=641)	Postpartum Hemorrhage (n=628)
	Mean (SD)	Mean (SD)	Mean (SD)	Mean (SD)	Mean (SD)
All physicians					
General practice (n=633)	53.9 (15.7)	57.3 (17.4)	59.0 (18.2)	48.5 (16.9)	54.9 (14.8)
Pediatricians (n=1,005)	–	–	63.2 (16.9)	56.2 (16.7)	–
Internal medicine (n=910)	58.7 (15.7)	66.2 (15.8)	56.5 (15.2)	45.4 (13.5)	60.3 (18.5)
Cardiologists (n=270)	55.9 (15.0)	69.2 (14.5)	–	–	–
OB/GYN (n=637)	–	–	51.7 (16.2)	54.1 (16.0)	64.0 (15.4)
Other physicians (n=45)	53.7 (16.2)	53.4 (17.9)	52.7 (12.4)	41.9 (17.3)	49.5 (6.9)
Midwives (n=353)	–	–	–	–	55.1 (17.0)

Abbreviations: CPV, Clinical Performance and Value; NCD, noncommunicable disease; OB/GYN, obstetricians/gynecologists; SD, standard deviation.

### Gaps in Clinical Care

This study identified specific issues of clinical concern that urgently suggest the need for remediation and follow-up measurement. In some countries, diagnosis of AMI was missed more than half (67%) the time. While aspirin is affordable and widely available in the countries studied, it was prescribed less than 40% of the time when indicated for AMI. The one exception was Russia, where aspirin was used appropriately 80% of the time. Similarly, cholesterol- and blood pressure-lowering drugs were used correctly less than 40% of the time when indicated.

Diagnosis of acute myocardial infarction was missed 67% of the time.

In the case of a newborn with birth asphyxia, only 32% of providers reported they would check for an open airway—universally poor across all countries in the study. Oxytocin, used for controlling postpartum hemorrhage, was prescribed only 64% of the time, although this figure masks cross-country differences. Georgia and Kazakhstan had oxytocin prescription rates above 70%, but for Albania, Armenia, and Tajikistan, this rate was below 60%.

Only 32% of providers reported they would check for an open airway in the case of birth asphyxia, and oxytocin was prescribed only 64% of the time to control postpartum hemorrhage.

Important gaps in care existed in the workup of patients, although not all providers performed poorly in all areas and not all areas of care were poor. For example, across all countries, 84% of providers, on average, identified the need to counsel patients with diabetes on proper diet and 75% of all providers, on average, ordered a chest x-ray in their neonatal cases to evaluate them for pneumonia. But there were important differences between countries; for example, neonatal x-rays to evaluate pneumonia were ordered by 91% of providers in Armenia but only by 64% of providers in Tajikistan. However, with few exceptions, providers did not use the tools (such as laboratory testing and imaging studies) available to appropriately work up the patient or monitor progress. Monitoring urine output for neonatal pneumonia cases was virtually nonexistent at 3%, with monitoring vital signs for these patients somewhat better at 17%. Fewer than half of the providers mentioned ultrasound—needed to evaluate postpartum hemorrhage—or vital signs for monitoring birth asphyxia.

Disaggregating quality of care scores by clinical domains—history taking, physical examination, laboratory and imaging workup, diagnosis, and treatment—unmasked a decay in quality of care across the patient interaction. The data showed that as providers progressed from history taking and physical examination (with CPV vignette averages above 60%), which center on collecting data, to later domains of testing and diagnosis, which involve making judgments about the data collected, scores declined. Treatment scores, the last domain in the CPV vignette encounter, were below 50% in all cases, with the exception of multiple NCD risk factors (average 66%).

There was a decay in quality of care across the patient interaction, with scores declining as providers progressed from history taking and physical examination to testing and diagnosis.

### Structural Quality Measures

Facilities participating in this study typically had quality structure scores less than 50% for most measures, with large hospitals having significantly better infrastructure and operational scores than PHC clinics (Table 6) (*P*<.01). Urban facilities typically had higher structural scores than rural facilities (*P*<.01), with differences ranging from 2% to 5%.

**TABLE 6.** Health Care Structural Quality Factors by Country and Facility Type

	Country and Facility Type											
	Albania		Armenia		Georgia		Kazakhstan		Kirov, Russia		Tajikistan	
	Total	Hospital	Total	Hospital	Total	Hospital	Total	Hospital	Total	Hospital	Total	Hospital
Average catchment	29,555	78,204	15,649	38,300	13,225	39,569	N/A	N/A	14,105	17,565	50,693	119,048
Average no. of full-time physicians	24	60	22	52	32	76	N/A	N/A	35	48	36	61
Average no. of full-time nurses	38	104	50	132	30	83	N/A	N/A	119	174	84	207
Infrastructure items												
Controls/operates pharmacy (%)	45%	92%	95%	100%	49%	100%	N/A	N/A	30%	42%	17%	37%
Owns transportation (%)	33%	100%	46%	83%	26%	69%	N/A	N/A	86%	96%	43%	72%
Medical equipment and supplies in working condition (% out of 54 items)	35%	60%	48%	75%	40%	72%	N/A	N/A	43%	56%	34%	53%
Laboratory tests available (% out of 35 items)	45%	58%	59%	73%	70%	72%	N/A	N/A	75%	77%	54%	60%
Medications in stock (% out of 43 items)	35%	52%	42%	54%	18%	45%	N/A	N/A	10%	15%	28%	44%
No. of ambulances with defibrillators	20.8	27.2	1.1	1.5	1.4	1.8	N/A	N/A	1.2	1.3	0.4	0.6
No. of computers in working condition	5.8	18.2	5.6	14.6	9.4	23.8	N/A	N/A	27.3	37.6	2.6	6.7
Operational items												
Committees/bodies at the facility												
*Quality assurance/review committee*	15%	31%	59%	94%	27%	63%	N/A	N/A	44%	60%	27%	46%
*Morbidity and mortality conference or committee*	12%	33%	30%	67%	18%	57%	N/A	N/A	48%	65%	52%	93%
*Infection control committee*	11%	36%	33%	60%	22%	59%	N/A	N/A	40%	53%	56%	76%
*Pharmacy or therapeutics committee*	16%	81%	56%	81%	16%	47%	N/A	N/A	9%	11%	10%	17%
Written guidelines for treatment and management of various conditions (% out of 18)	58%	60%	66%	60%	55%	44%	N/A	N/A	79%	81%	34%	36%
Able to pull patient chart based on name (%)	77%	72%	98%	98%	55%	65%	N/A	N/A	34%	40%	48%	52%
Have system to track patients who require chronic care but miss follow-up (%)	39%	21%	58%	29%	46%	45%	N/A	N/A	86%	83%	68%	46%

## DISCUSSION

The findings from this study suggest that the 6 ECA countries studied are challenged by poor quality clinical care, regardless of practice setting, specialty, or facility type. Like other settings worldwide, we found high quality of care is the exception and not the norm.^5^^,^^17^^,^^20^^,^^36^^,^^50^ Intra-country quality, as measured by performance on CPV vignettes, varied by as much as 20 percentage points, but within-country quality varied by as much as 65 percentage points. Still, within each country, many individual practitioners performed well. These results provide a comparative basis describing what *can* be achieved (i.e., the highest performers), even under difficult conditions.

The wide variation in CPV scores across all countries indicates that as many as 1 in 6 providers may deliver worrisomely low-quality levels of care. Even the typical score, which for all 5 conditions evaluated in the CPV vignettes was midrange (45% to 55%), indicates that on CPV cases providers carried out just over half of the patient care criteria recommended by guidelines and practice standards.

By contrast, in every country, a considerable proportion of practitioners *delivered high-quality care*. Among physicians, 11% of the sample scored greater than 80%, suggesting that good care quality is possible even within the constraints of a region's health systems. The presence of high performers is notable among both physicians and midwives.

Good quality of care is possible even within the constraints of a region's health systems.

The lower quality of care scores, as measured by CPV vignettes, observed in primary health care clinics and rural areas compared with hospitals demonstrates a need to target quality improvement efforts geographically, particularly toward primary care diagnosis and treatment.^51^^–^^53^ This appears to be especially important for reaching the poor who are likelier to live in rural areas and access care through primary care providers.^54^

Performance by midwives on CPV vignettes was significantly higher in Kazakhstan and Tajikistan, where the regulatory environment enables independent practice by this profession. Improved training and structural support for midwives in other countries would potentially help promote care quality improvements in their countries.

The notable decay in quality across the care encounter (from history taking to diagnosis) raises important questions about providers' ability to accurately diagnose and treat conditions.^55^ Furthermore, the conditions with the lowest scores—multiple NCD risk factors, birth asphyxia, and postpartum hemorrhage—are conditions for which low-cost, widely available treatments can significantly reduce mortality. The low CPV vignette scores identify specific deficiencies that, if remedied, would rapidly improve quality of care and outcomes.

The conditions with the lowest scores were multiple NCD risk factors, birth asphyxia, and postpartum hemorrhage, conditions for which low-cost, widely available treatments can significantly reduce mortality.

### Policy Implications of CPV Vignettes

A number of policy levers, such as the Balanced Score Card,^56^ accreditation,^57^ and Pay for Results,^6^^,^^34^ are generally available to improve quality of care. Effective serial monitoring has become a prerequisite for demonstrating the success (or failure) of these or other policies over time.^58^ Adding public reporting of quality measures would help to improve quality by increasing accountability,^58^ promoting improvement in the health status of the populations in the 6 ECA countries,^59^ and lowering costs.^60^ In addition to supporting public reporting, serial CPV vignette measurement can be used to monitor the effectiveness of any initiative introduced to improve the process quality of care, as well as potential links to performance incentives.

### Benefits of the CPV Vignette Methodology

Few large-scale quality of care assessments have been undertaken, in part, due to the absence of effective measurement tools that provide robust, reliable, and case-mix adjusted results across diverse economic, cultural, and social settings. While all process of care measures have limitations, this study shows that quality can be measured widely and affordably using CPV vignettes.

This study shows that quality can be measured widely and affordably using CPV vignettes.

There have been other evaluations of a similar scale and at both inter- and intra-country levels, such as the Service Provision Assessment (SPA).^24^^,^^31^ However, the process of care evaluation used in the SPA is limited compared with this study, as CPVs measure the entire clinical process of care without requiring a clinical evaluator or introducing the bias that occurs with direct observation.

Implementation of this study demonstrates that large-scale inter- and intra-country process of care quality evaluations are only possible with active local participation, a multidisciplinary team, and attention to data collection training. This study enlisted community members for training staff, piloting data collection, and conducting ongoing quality assurance checks, paying particular attention to supervision and data consistency.

The study, conducted in 6 ECA countries, measured quality of care among 3,584 physicians and 384 midwives in 1,039 facilities, making it the largest cross-national comparison of quality undertaken in this region. The process of measuring the quality of clinical services at cross-national scale and providing benchmarks was done relatively quickly, making quality of care measurement a powerful policy opportunity for improving health.

### Limitations

This study was conducted in 5 countries and 1 large province of the Russian Federation and thus cannot be considered representative of the entire ECA region.

A potential concern is that CPVs do not reflect actual provider *practice* and are instead only measuring *knowledge*,^61^ which is important but potentially different than practice. CPV vignettes, unlike other vignettes, have been validated in the United States against actual practice. They proved to correlate closely with provider behavior and are therefore a useful measure of quality of care.^6^^,^^29^^,^^27^^,^^62^^–^^65^ In some cases, where procedural intervention is required (e.g., a surgical technique, psychotherapy, or pediatric care), CPVs cannot be validated with standardized patients. These exceptions notwithstanding, the published validation studies have shown that CPVs correlate well with measures using standardized patients, the gold standard for measurement of clinical performance in some of the clinical cases we studied (e.g., pneumonia, NCD risk factors, AMI),^6^^,^^29^^,^^27^^,^^63^^,^^66^^–^^68^ with the notable exception of postpartum hemorrhage and neonatal asphyxia.^69^ However, validation can be viewed as whether the measurement is responsive and able to explain better outcomes. Chief among these studies is the experimental evidence that when CPV scores improve, not only does actual practice change but so do health outcomes of patients cared for by those providers.^34^ This evidence, summarized in a literature review of the best measures of clinical practice, concluded that CPV vignettes can be an effective way to assess the quality of care across facilities and large numbers of providers.^62^ CPVs have been deployed at an affordable cost in a variety of clinical practice settings around the world.^6^^,^^8^^,^^30^ The authors note that similar validation against standardized patients in low- and middle-income countries would enhance earlier validation work, although performing such studies could prove difficult, if not impossible.

While not done in this study, serial CPV measurement with feedback of results to providers has been demonstrated in a number of settings to improve care quality and health outcomes in the population.^70^ This study, with its 1 round of measurement, could not demonstrate the impact of measurement on practice. Ultimately, quality measurement must be done serially and go beyond benchmarking to motivating changes in practice. Every country needs to link changes in knowledge and practice to changes in practice and health status. This has already been done using CPV vignettes in some countries.^58^

While CPVs are comprehensive measures of the specific clinical care processes, they do not measure structural or other elements of care. For example, this article does not address patient satisfaction as a critical indicator of quality nor the adequacy of drug supplies or of medical supervision. Interestingly, given the central nature of clinical care practice to all dimensions of quality, a review conducted by Doyle and colleagues showed a consistent link between technical quality and patient satisfaction.^71^ In our own experimental studies, using the CPV methodology, we have found that as CPV scores improve, so does patient satisfaction.^72^

Finally, this was a descriptive study. It did not determine how much of the gaps in measured performance could be attributed to modifiable vs. non-modifiable factors. Ideally, this would be done experimentally or prospectively in a follow-on to this large-scale study.

## CONCLUSIONS

This study provides a comprehensive and detailed baseline picture of health care quality across 3 clinical conditions in 6 ECA countries. While the data show that excellent quality of care is possible in all of these countries and in all types of facilities, it also provides an alarming picture of poor and variable quality, as measured by CPV vignettes. National and cross-national measuring and benchmarking the process of care among peers—if done serially—could spur quality improvement efforts that raise overall quality of care and decrease clinical variability.^73^^,^^74^

## Supplementary Material

Supplement

## Notes

**Peer Reviewed**
