# Supplementary material for: Large-Scale Evaluation of Quality of Care in 6 Countries of Eastern Europe and Central Asia Using Clinical Performance and Value Vignettes
Source: Glob Health Sci Pract. 2017 Sep 27;5(3):412–29. doi: 10.9745/GHSP-D-17-00044 (PMC5620338; doi:10.9745/GHSP-D-17-00044)
Supplement: Supplement [file GHSP-D-17-00044_index.html]

Supplement to Large-Scale Evaluation of Quality of Care in 6 Countries of Eastern Europe and Central Asia Using Clinical Performance and Value Vignettes | Global Health: Science and Practice

## Supplemental material

- Text s01, PDF - Text s01, PDF
