# Supplementary material for: Large-Scale Evaluation of Quality of Care in 6 Countries of Eastern Europe and Central Asia Using Clinical Performance and Value Vignettes
Source: Glob Health Sci Pract. 2017 Sep 27;5(3):412–29. doi: 10.9745/GHSP-D-17-00044 (PMC5620338; doi:10.9745/GHSP-D-17-00044)
Supplement: Supplement [file 17-00044-Peabody-Supplement.pdf]

Peabody JW, DeMaria L, Smith O, Hoth A, Dragoti E, Luck J. Large-scale evaluation of quality of care in 6 countries of Eastern Europe and Central Asia using Clinical Performance and Value vignettes. *Glob Health Sci Pract*. 2017;5(3). https://doi.org/10.9745/GHSP-D-17-00044

**SUPPLEMENT.** Scoring Criteria for the Neonatal Pneumonia Clinical Performance and Value Vignette and Results Comparing a High- and Low-Performing Physician

| Physician A (High-Performing) | | | Physician B (Low-Performing) | |
|---|---|---|---|---|
| **Asked** | **Omitted** | **Scoring Item** | **Asked** | **Omitted** |
| **History** | | | | |
| X | | Onset and severity of symptoms | X | |
| X | | Duration and course of symptoms | X | |
| X | | Associated fever, seizures, rash, somnolence, etc. | | X |
| X | | Feeding history | X | |
| X | | Hydration status | X | |
| | X | Gestational age | | X |
| X | | Gravidity and parity | | X |
| X | | Recent history of infection or fever in mother | | X |
| X | | Labor duration and specifics of delivery | | X |
| X | | Maternal sedation in labor | | X |
| X | | Hours of ruptured membranes or if there is meconium staining | | X |
| X | | Need for resuscitation at birth | | X |
| | X | Apgar scores | | X |
| | | Other maternal medical problems or medications | | X |
| **Physical Examination** | | | | |
| X | | Vital signs | X | |
| X | | Oxygenation, skin color | X | |
| X | | Auscultation of lungs | X | |
| X | | Auscultation of heart | X | |
| X | | Capillary refill time | | X |
| | X | Palpation/auscultation of the abdomen | | X |
| **Workup** | | | | |
| X | | Complete blood count with differential | X | |
| X | | Blood culture | X | |
| X | | Glucose | | X |
| X | | Spinal tap | | X |
| X | | Chest x-ray | X | |

Peabody JW, DeMaria L, Smith O, Hoth A, Dragoti E, Luck J. Large-scale evaluation of quality of care in 6 countries of Eastern Europe and Central Asia using Clinical Performance and Value vignettes. *Glob Health Sci Pract*. 2017;5(3). https://doi.org/10.9745/GHSP-D-17-00044

| **Physician A (High-Performing)** | | | **Physician B (Low-Performing)** | |
|---|---|---|---|---|
| **Asked** | **Omitted** | **Scoring Item** | **Asked** | **Omitted** |
| **Diagnosis** | | | | |
| Neonatal sepsis with pneumonia and meningitis | | Primary diagnosis | Pneumonia, community-acquired | |
| Moderate-severe | | Severity | Moderate | |
| **Treatment** | | | | |
| X | | Admit to hospital | X | |
| X | | Supplemental oxygen by face mask | | X |
| X | | Intravenous (IV) glucose 10% in 0.18 normal saline | | X |
| X | | IV antibiotics: ampicillin+aminoglycoside or IV aminoglycoside+expanded spectrum penicillin | X | |
| X | | Monitor vital signs | | X |
| | X | Monitor occipitofrontal circumference | | X |
| X | | Repeat blood cultures after 24–48 hours | | X |
| X | | Repeat lumbar puncture after 24–48 hours of initiating antibiotics | | X |
| **Total CPV Score** | | | | |
| **31/35=89%** | | | **14/35=40%** | |
